# Charting Plausible Futures for Diabetes Prevalence in the United States: A Role for System Dynamics Simulation Modeling

**Published:** 2007-06-15

**Authors:** Bobby Milstein, Andrew Jones, Jack B Homer, Dara Murphy, Joyce Essien, Don Seville

**Affiliations:** Syndemics Prevention Network, Centers for Disease Control and Prevention; Sustainability Institute, Asheville, NC; Homer Consulting, Voorhees, NJ; Centers for Disease Control and Prevention, Atlanta, Ga; Rollins School of Public Health, Emory University, Atlanta, Ga; Sustainability Institute, Hartland, Vt

## Abstract

**Introduction:**

*Healthy People 2010* (*HP 2010*) objectives call for a 38% reduction in the prevalence of diagnosed diabetes mellitus, type 1 and type 2, by the year 2010. The process for setting this objective, however, did not focus on the achievability or the compatibility of this objective with other national public health objectives. We used a dynamic simulation model to explore plausible trajectories for diabetes prevalence in the wake of rising levels of obesity in the U.S. population. The model helps to interpret historic trends in diabetes prevalence in the United States and to anticipate plausible future trends through 2010.

**Methods:**

We conducted simulation experiments using a computer model of diabetes population dynamics to 1) track the rates at which people develop diabetes, are diagnosed with the disease, and die, and 2) assess the effects of various preventive-care interventions. System dynamics modeling methodology based on data from multiple sources guided the analyses.

**Results:**

With the number of new cases of diabetes being much greater than the number of deaths among those with the disease, the prevalence of diagnosed diabetes in the United States is likely to continue to increase. Even a 29% reduction in the number of new cases (the *HP 2010 *objective*)* would only slow the growth, not reverse it. Increased diabetes detection rates or decreased mortality rates — also *HP 2010* objectives — would further increase diagnosed prevalence.

**Conclusion:**

The *HP 2010* objective for reducing diabetes prevalence is unattainable given the historical processes that are affecting incidence, diagnosis, and mortality, and even a zero-growth future is unlikely. System dynamics modeling shows why interventions to protect against chronic diseases have only gradual effects on their diagnosed prevalence.

## Introduction

In each of the past three decades, national public health objectives in the United States have been set 10 years into the future and published as health objectives for the nation (1-3). These objectives define specific numerical targets for reductions in most major health problems as well as for increases in the prevalence of health-promoting behaviors. J. Michael McGinnis, MD, a chief architect of the objective-setting enterprise, asserts that, "Of the broad range of governmental responsibilities in public health, perhaps none is more fundamental than the obligation to provide perspective and direction to guide health programs along a productive course — the agenda-setting function" (4).

Considering the widespread use and significance of the *Healthy People* (*HP*) objectives for planning and evaluating public health work at all levels of practice, health care practitioners may expect national health objectives to be feasible, that is, to be achievable within the specified time frame. However*, HP* objectives may not always meet this feasibility standard (5). The objectives for 2010, in particular, were set on the basis of a policy goal of eliminating health disparities among racial and ethnic groups. Consequently, planners used a "better than the best" approach wherein each objective was set at a level better than that of the "best" (i.e., most healthy) racial or ethnic group. That approach advanced health equity as an important philosophical ideal, which, in turn, generated an ambitious aspiration for health policy-making. But it may not have yielded, in all cases, objectives that are achievable and compatible with other public health objectives. In addition, the practice of conducting midcourse reviews and periodic evaluations of progress toward meeting *HP* objectives may convey the impression that the numerical targets are actually achievable by 2010 and are therefore meaningful referents for assessing progress (6,7).

Questioning whether long-range objectives can be met raises additional questions about the analytic procedures that guide objective-setting itself, which is a complicated dimension of public health science that is still poorly understood. In this article, we illustrate how system dynamics (SD) simulation modeling can inform the development and understanding of national public health objectives. Specifically, we use an SD model to 1) interpret the historic prevalence record of diagnosed diabetes (as used throughout this article, *diabetes* refers to diabetes mellitus, types 1 and 2) in the United States; and 2) anticipate the future prevalence of diabetes through 2010 under various scenarios.

**Figure 1:**
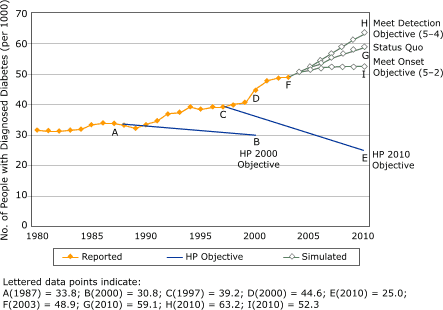
Diagnosed prevalence of diabetes per 1000 total population, United States, 1980–2003 (8), with *Healthy People 2000* and *Healthy People 2010* objectives (2,3), and simulation model output, for 2003–2010 (13).

Figure 1 displays the observed trend in the prevalence of diagnosed diabetes (diagnosed prevalence) per 1000 population from 1980 through 2003 (8). It also illustrates the two paths that *HP 2000* and *HP 2010* objectives indicate for diabetes prevalence (2,3). In 1990, after three decades in which diabetes prevalence increased (9), the *HP 2000* baseline was set on the basis of 1987 data (point A), and the *HP 2000* objective called for an 11% reduction in prevalence by 2000 (point B). Instead, diagnosed prevalence increased by 33% between 1987 and 2000 (from point B to point D). The official final review of *HP 2000* showed that prevalence "moved away from target" (10) by 367% (calculated by comparing the D-to-B gap with the A-to-B target decrease).

**Table:** 

Figure 1 graphs the trend of diabetes prevalence between 1980 and 2003, along with *Healthy People* objectives for 2000 and 2010 as well as simulation results from three experiments discussed in the narrative. The x axis is calibrated for years between 1980 and 2010. The y axis indicates the number of people with diagnosed diabetes per 1000 population. It is labeled in increments of ten. Three prevalence lines are graphed: (1) reported, (2) *Healthy People* objectives, and (3) simulated scenarios. Figure 1 gives the following data points:
Reported prevalence: 1987, 33.8; 1997, 39.2; 2000, 44.6; in 2003, 48.9 *Healthy People* objective: 2000, 30.8; 2010, 25.0 Simulated: in 2010, 59.1 under the status quo scenario; 63.2 under meets the *Healthy People* detection objective 5-4 scenario; 52.3 under the meets *Healthy People* onset objective 5-2 scenario.

The *HP 2010* objective, which was based on 1997 data, called for an even more ambitious 38% reduction in diabetes prevalence, from 39.2% to 25.0% (point C to E). But again, surveillance data revealed a worsening trajectory. From 1997 to 2003, diabetes prevalence increased another 25% (point C to point F), making the 2010 objective even more unattainable.

What accounts for these discrepancies between prevalence objectives and actual prevalence data? Are they due to poor performance of the overall national health protection strategy, which includes an array of separately focused programs and policies (11)? Or are they perhaps the result of a flaw in how the numerical targets are derived? If the latter is the case, what is a more plausible estimate of the actual trajectory of U.S. diabetes prevalence through 2010?

## Methods

Members of the Centers for Disease Control and Prevention (CDC) Diabetes System Modeling Project sought to answer these questions by conducting a series of simulation experiments using an existing SD model designed specifically to explore the population dynamics of diabetes among adults in the United States (12,13). The model was designed to explore the incremental effects various possible policy interventions could have on the burden of diabetes. To achieve this result, the SD model, unlike other diabetes models (for example, a Markov model by Honeycutt et al [14]), comprehensively accounts for a chain of population flows that begins when a person becomes at risk for diabetes and continues through initial onset, diagnosis, progression, and death. Such breadth of scope allows the SD model to anticipate nonlinear changes in variables, such as the incidence rate, that narrower models would miss (22).

We developed the SD diabetes model using well-established techniques for model formulation and testing (15-21). Data from the National Health Interview Survey, the National Health and Nutrition Examination Survey (NHANES), the Behavioral Risk Factor Surveillance System, the U.S. Census Bureau, and publications in the scientific literature provided the empirical foundation for parameter selection and estimation. We were able to draw some parameter estimates directly from available information, and we obtained others through a process of historical curve-fitting analogous to statistical regression. (For more detail on the sources and methods used in determining the parameters for the SD diabetes model, see references 13 and 22.)

### Structure of the diabetes system

**Figure 2:**
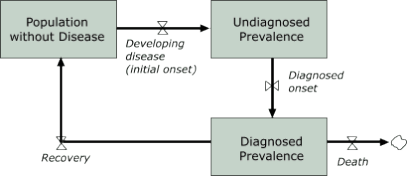
Generic stock-and-flow structure for diagnosed prevalence of a disease.

The SD diabetes model specifies how population groups accumulate in several *states* of health (e.g., prediabetes, uncomplicated diabetes, complicated diabetes) along with the *rates* at which people flow from one state to another. The full model contains many such states and rates (13); however, in Figure 2 we show only a simplified and generic view for explanatory purposes.

**Table:** 

Figure 2 is a stock-and-flow diagram describing the incidence, diagnosis, and prevalence of a disease in a population. It includes three mutually exclusive population stocks as well as the related rates of change among them.
The first stock, labeled *Population Without Disease*, has a single outflow for the rate of *People Developing Disease* (or *initial onset*), which in turn, is the only inflow into the stock of *Undiagnosed Prevalence*. *Undiagnosed Prevalence* has a single outflow rate for *Diagnosed Onset*, leading to the final stock of *Diagnosed Prevalence*.
There are two outflows from *Diagnosed Prevalence*, one for deaths and another for recovery, which returns people back to the *Population without Disease*.

Figure 2 depicts a generic stock-and-flow structure that can be used to illustrate the diagnosed prevalence for any disease. One may think of the box labeled *diagnosed prevalence* as a bathtub in which the water level represents the number of people who have been diagnosed with a disease (23). The rate at which a condition is diagnosed, *diagnosed onset*, is analogous to the rate at which water flows into the bathtub. The rates of *recovery* or *death* for people with diagnosed disease are analogous to the rates at which water flows out of the bathtub through two separate drains. As Figure 2 illustrates, all changes in diagnosed prevalence must be accounted for by changes in these related flows. (For a complete accounting, the flows of births, migration, and recovery among the undiagnosed, as well as deaths among those without the disease and deaths among the undiagnosed, would be needed, but for clarity these are not depicted in Figure 2.)

Following is a summary of how the generic elements of Figure 2 relate specifically to diabetes:

- *Diagnosed prevalence*: Figure 1 provides historical data for 1980 through 2003. In 2000, about 12.0 million people of all ages in the United States had diagnosed diabetes; of these 12.0 million people, 98% were adults aged 20 years and older. This percentage translates to about 4.4% of the total population and 6.0% of the adult population (14,24).
- *Diagnosed onset*: About 880,000 people were newly diagnosed with diabetes in 1997, and that figure rose to 1.1 million by 2000. Of these 1.1 million people, more than 96% were adults. This percentage translates to a diagnosis rate among the adult population of about 5.2 per 1000 in 2000 (14,24).
- *Recovery*: Recovery is a significant factor in prevalence calculations for many acute illnesses; however, in diabetes, as for all chronic diseases without a cure, it is not a factor.
- *Deaths among people with diagnosed diabetes*: Diabetes, like many other chronic diseases, has a relatively low annual death rate. In 2000, of the 12.0 million people with diagnosed diabetes, about 500,000 (4.2%) died (14). Of these deaths, 213,000 (a rate of 1.8% per year among Americans with diabetes) were related to complications of the disease (24).
- *Undiagnosed prevalence*: Since 1976, researchers have tested blood glucose levels in random samples of adults without diagnosed diabetes who were participants in the periodic NHANES (9) to determine if they had diabetes. By dividing the number of people found to have diabetes by the total number of people tested, researchers estimated the fraction of Americans with undiagnosed diabetes for each of the following NHANES periods: 1976–1980: 38%; 1988–1994: 36%; and 1999–2000: 29% (25).
- *Initial Onset*: There is no actual measure of the rate of initial onset of diabetes (i.e., the number of people per year who develop diabetes) as opposed to the rate of diabetes diagnosis. However, we estimated the initial onset rate by combining the data described above on diagnosed prevalence, undiagnosed prevalence, and death using the causal logic of Figure 2. According to our estimates, in 2000, 1.25 million U.S. adults experienced the initial onset of diabetes, a rate of 6.0 people per 1000.
- *Population without diabetes*: This category includes people with normal blood glucose levels and those with prediabetes, a condition in which levels are moderately elevated (26). According to estimates based on blood test data from NHANES 1988–1994, about 40% of Americans aged 40 to 74 years had prediabetes (24,26). We extrapolated this figure to the rest of the adult population, using historical data on age-specific diabetes incidence (14), to estimate differences in prediabetes prevalence between people aged 18 to 39 years and those aged 75 and older. Projecting forward in time, we estimated that at least 52 million (25%) of Americans aged 18 and older had prediabetes in 2000.

### Exploring scenarios for the future

The SD model employed in our simulations tracks the flows and accumulations of people with normal blood glucose levels, undiagnosed or diagnosed prediabetes, undiagnosed or diagnosed diabetes without complications, and undiagnosed or diagnosed diabetes with complications. In the model, we specify key factors — some of them potentially amenable to policy intervention — that may change over time and that affect the model's population flows. These variable policy factors include the prevalence of obesity (i.e., the leading modifiable risk factor for diabetes); the prevalences of glycemic screening, prediabetes management, and diabetes management; as well as the percentage of the population with access to preventive care (13). A *scenario* involves specified future values for each variable factor. The model can then simulate the consequences of any given scenario for future trajectories of diagnosed prevalence and other measures of disease burden.

## Results

### A status quo scenario

Beginning in 2004, results from the first simulation experiment focus on a *status quo* future, in which we assumed no further changes in the scope or effectiveness of prevention, detection, or management efforts or in the prevalence of obesity. In Figure 1, the line marked *status quo* (from point F to G) shows the projected prevalence of diagnosed diabetes through 2010 under these assumptions. Diagnosed prevalence is projected to rise throughout this period because the inflow of people with newly diagnosed diabetes is projected to exceed the rate at which people are dying. Under this scenario, the prevalence of diagnosed diabetes is projected to increase 21%, from 48.9 per 1000 in 2003 to 59.1 per 1000 in 2010 (point F to G).

A straightforward comparison of the estimates of inflow (diagnosis) and outflow (death) explains why the upward trend in diabetes prevalence, which began around 1990, will not soon abate. If the diagnosed onset rate in 2000 of approximately 1.1 million cases per year and the death rate of about 500,000 per year were to stay the same, we projected that the diagnosed prevalence will continue to increase. Although the model suggests that this gap between inflow and outflow is gradually closing, the inflow of diagnosed diabetes onset would have had to drop substantially (e.g., by about 50% in 2006) just for diagnosed prevalence to stop increasing, let alone to begin decreasing.

### Accounting for program and policy interventions   

Our SD model reveals certain insights about the long-term effects of interventions to reduce onset, boost detection, and better manage diabetes. For example, aside from reducing prevalence, another *HP 2010* objective calls for the percentage of people with diagnosed diabetes to increase from 68% to 80% (Objective 5-4) (3). Such an increase in diagnoses would, in terms of Figure 2, increase the inflow of people diagnosed with the disease. Thus, the prevalence of diagnosed diabetes would also increase. Figure 1 quantifies the effect of this scenario as the difference between points H and G (i.e., 63.2 vs 59.1 in the year 2010).

Another *HP 2010* objective calls for an 11% reduction in the diabetes-attributable death rate (Objective 5-6) (3), a result presumably to be achieved through improved disease management. As Figure 2 indicates, a reduction in deaths would also increase diagnosed prevalence because more people with the disease would remain alive. (Figure 1 does not display a curve for this scenario because it overlaps the status quo line [i.e., 59.3 vs 59.1 in the year 2010]).

The inconsistency in these *HP 2010* objectives for diabetes is clear: meeting the objectives for increasing the diagnosis rate or decreasing the mortality rate would tend to increase the prevalence of diagnosed diabetes.

One type of public health intervention that could possibly reduce the diagnosed prevalence of diabetes would be one aimed at reducing the initial onset rate. *HP 2010* calls for a 29% reduction in the number of new diabetes diagnoses per 1000 (Objective 5-2) (3), presumably to be achieved through enhanced efforts to detect and manage prediabetes. This effort would, perhaps, be combined with efforts to reduce the prevalence of obesity in the general population. A reduction in the inflow resulting from diagnosed disease onset is clearly a move in the right direction because it leads to a lower diagnosed prevalence than would be the case under the status quo (i.e., without an intervention to reduce disease onset). But a reduction in diagnosed prevalence *relative to the status quo* is not the same as an absolute reduction over time — an actual reversal of growth. We described previously how a straightforward comparison of the inflow and outflow rates in Figure 2 indicates that a reduction in the onset of diagnosed diabetes on the order of 50% would be required to halt the growth in diagnosed prevalence. However, the question still remains: to what extent could a significant reduction in onset at least slow the growth of diagnosed prevalence?

To address this question, we simulated an intervention begun in 2003 that would reduce the rate of diabetes onset 29% below its 1997 level by 2010. In this scenario, as shown in Figure 1, the prevalence of diagnosed diabetes increases by 7% (from F to I) per 1000 population from 2003 to 2010 as compared with increasing by 21% (from F to G) in the status quo scenario (i.e., 52.3 vs 59.1 in the year 2010). This slower growth in the number of people with diagnosed diabetes certainly would improve the overall disease picture, but it would not yield a decline in diabetes prevalence.

The simulation modeling thus helps quantify what the stock-and-flow logic of Figure 2 and previously described numerical analysis suggested: that the *HP 2010* target of a 29% reduction in the rate of diabetes onset is too modest a reduction to achieve the desired reduction in prevalence and can only slow its growth.

The SD model can also be used to explore more extreme possibilities. For example, what would happen if initial onset of undiagnosed diabetes had dropped suddenly to zero during 2004? Even under this impossible-to-achieve scenario, diagnosed prevalence would decrease by only 14% from 2003 to 2010 (data not shown). This decrease is relatively small partly because cases of diabetes will continue to be diagnosed during this period even though initial onset has ceased, and partly because of the relatively low death rate among people with diabetes (about 4% per year). Thus, even this most optimistic scenario of a 14% reduction in diagnosed prevalence of diabetes during 2003 through 2010 falls far short of the 38% objective of *HP 2010*.

## Discussion

### Charting plausible paths

Findings from our study indicate that the *HP 2010* objective for reducing diagnosed diabetes prevalence by 38% will not be achieved — not because of ineffective or underfunded health protection efforts but because the objective itself is unattainable. Moreover, if current investments in diabetes screening and disease management continue to succeed in diagnosing a greater number of people and in enabling people to live longer with the disease, then diagnosed prevalence will move still farther away from the *HP 2010* target.

In setting long-range numerical targets for health objectives, particularly those that may be viewed as intervention outcomes, it is important to recognize that the diagnosed prevalence metric is subject to misinterpretation and to unrealistic expectations for two basic reasons:

- The task of setting plausible prevalence objectives requires an understanding that the growth in prevalence of many chronic diseases can, at best, be slowed, and it can be reversed only gradually. This is because the number of deaths from chronic disease is small relative to increases resulting from disease onset (perhaps, as in the case of diabetes, because of a decades-long increase in the at-risk population), and there is no significant reduction as a result of people recovering from chronic diseases. Therefore, the task of reducing prevalence is comparable to attempting to return a fast-moving train to a station that it passed miles back: the first step is to slow the train down, not to reverse its direction.
- As a result of successful interventions to increase disease detection and management, people are living longer with chronic diseases rather than dying prematurely. But by increasing detection and extending life, such interventions also have the effect of increasing diagnosed prevalence of these diseases. The only practical way of slowing the growth in diagnosed prevalence is through health protection programs that reduce the rate of disease onset. However, onset must not only decline. It must fall far enough to more than offset the increase in disease prevalence resulting from improved detection and management. It will be impossible to set achievable prevalence-reduction objectives unless this relationship is taken into consideration.

If prevalence objectives are to be attainable within their specified time frame, it is important first to recognize what the disease trajectory would be under status quo assumptions and then to factor in the effects of any planned interventions, recognizing that measurable success in one area (e.g., an increase in the percentage of cases diagnosed) may reduce apparent progress in others (e.g., decreased prevalence). In the case of diabetes, we found in our simulation experiments that under current conditions, that is, without any new interventions, diagnosed prevalence would increase 21% from 2003 to 2010. Proposed detection and management initiatives, if successful, would increase that number even further. If disease prevalence is to serve as a benchmark for assessing the performance of national public health interventions, then prevalence-reduction goals must account for the compounding effects of successful disease detection and management interventions.

### Recognizing the benefits of formal modeling

Simulation modeling helps improve our collective understanding of health and disease dynamics, and in turn supports the development of long-range objectives that are both achievable and mutually consistent. Such models enable planners and policy makers to explore for themselves the plausible short- and long-term consequences of historic trends and to compare the effects of alternative interventions before committing limited resources. For that reason, diabetes program planners in Vermont have worked with members of the CDC Diabetes System Modeling team to use the model described here as a support for their efforts to set plausible and internally consistent objectives for diabetes-related outcomes at the state level (28,29). Planners in Minnesota, California, Alabama, Tennessee, and Florida are currently exploring similar uses.

Without the reality checks available through formal simulation experiments, long-range target-setting may fall prey to the weaknesses of flawed and sometimes biased intuition (mental models) (17,23). Popular conceptions about how certain phenomena change over time may often fail to account for real-world sources of inertia and delay and may suggest that things can change more rapidly than is actually possible. The prevalence of a chronic disease like diabetes changes only gradually because, as noted above, people with such conditions die at a relatively slow rate, and there is currently no cure for these conditions. In this respect, chronic diseases are unlike many acute infectious diseases such as influenza or measles, whose victims do not linger in the disease condition for years but instead recover or die relatively quickly. For such acute diseases, the large outflow creates a close correlation between decreases in the rate of onset and in diagnosed prevalence. For chronic illnesses, however, decreases in onset rates do not correlate with immediate decreases in prevalence; instead, they correlate with prevalence increasing more slowly.

Those working to prevent and manage chronic diseases may use stock-and-flow diagrams to develop a clearer understanding of the characteristic dynamics of these diseases. In addition, simulation experiments may bring new insights to the task of setting realistic and achievable goals for the nation's health. That approach could help ensure that numerical objectives are mutually consistent and achievable within their stated time frames. The objectives may still be difficult to achieve in practice and in that sense may be aspirational. But even aspirational objectives can and should be crafted in a way that is consistent, logical, and feasible given the causal structure of the system and the historical processes under way, particularly those responsible for the pattern of increasing incidence and diagnosis, as well as declining mortality.

Although simulation models can help improve our understanding of chronic disease dynamics, they have several inherent limitations. All models are incomplete simplifications of reality, and their conclusions are affected both by structural boundaries and by the uncertainties of the data with which they are calibrated (29). Techniques such as boundary critique (30) and sensitivity testing (17,31) can be used to assess the extent to which models may be affected by these simplifications and uncertainties. In the case of the diabetes SD model, sensitivity testing suggests that the *magnitudes* of its simulated futures, such as those seen in Figure 1, are subject to some imprecision because of uncertainties about input parameters, but that the *directions* of change and thus our general findings are unaffected by these uncertainties.

Even with their inevitable imprecision and incompleteness, simulation models can enhance learning and decision making, and that is their primary purpose (17,32). These tools can improve our collective understanding about how interventions will affect health indicators over many years within the complex systems of cause and effect that shape the public's health.
